# Participant preferences for an Aboriginal-specific fall prevention program: Measuring the value of culturally-appropriate care

**DOI:** 10.1371/journal.pone.0203264

**Published:** 2018-08-31

**Authors:** Blake Angell, Tracey Laba, Caroline Lukaszyk, Julieann Coombes, Sandra Eades, Lisa Keay, Rebecca Ivers, Stephen Jan

**Affiliations:** 1 The George Institute for Global Health, University of New South Wales, Sydney, Australia; 2 The Poche Centre for Indigenous Health, Sydney Medical School, the University of Sydney, Sydney, Australia; 3 The University of Sydney, Menzies Centre for Health Policy, School of Public Health, Sydney Medical School, Sydney, New South Wales, Australia; 4 Baker IDI, Melbourne, Victoria, Australia; 5 School of Public Health & Community Medicine, University of New South Wales, Sydney, Australia; RWTH Aachen University Hospital, GERMANY

## Abstract

**Background:**

Culturally-specific services are central to efforts to improve the health of Aboriginal Australians. Few empirical studies have demonstrated the value of such services relative to mainstream alternatives.

**Objective:**

To assess the preferences and willingness to pay (WTP) of participants for attending a class and the relative importance of transport, cost and cultural-appropriateness in the choices made by participants.

**Design:**

A discrete choice experiment (DCE) was conducted alongside a study of a culturally-specific fall-prevention service. Attributes that were assessed were out-of-pocket costs, whether transport was provided and whether the class was Aboriginal-specific. Choices of participants were modelled using panel-mixed logit methods.

**Results:**

60 patients completed the DCE. Attending a service was strongly preferred over no service (selected 99% of the time). Assuming equivalent efficacy of fall-prevention programs, participants indicated a preference for services that were culturally-specific (OR 1.25 95% CI: 1.00–1.55) and incurred lower out-of-pocket participant costs (OR 1.19 95% CI 1.11–1.27). The provision of transport did not have a statistically significant influence on service choice (p = 0.57).

**Discussion and conclusions:**

This represents the first published DCE in the health field examining preferences amongst an Aboriginal population. The results empirically demonstrate the value of the culturally-specific element of a program has to this cohort and the potential that stated-preference methods can have in incorporating the preferences of Aboriginal Australians and valuing cultural components of health services.

**Note on terminology:**

As the majority of the NSW Aboriginal and Torres Strait Islander population is Aboriginal (97.2%), this population will be referred to as ‘Aboriginal’ in this manuscript.

## Introduction

Falls are a leading cause of injury amongst older Aboriginal Australians as they are in Indigenous and non-Indigenous communities around the world [1]. Fall-prevention programs are an evidence-based means of reducing the risk of older people experiencing a fall or to improve outcomes following a fall [2]. The effectiveness of such services has been shown repeatedly in the literature [3], however, the effectiveness of mainstream programs in reaching Aboriginal populations is unknown [4]. There is a documented underutilisation of primary care services by Aboriginal communities attributable to barriers to services including financial, cultural and geographic impediments to care [5, 6]. In spite of this, however, Aboriginal people have been shown to access existing aged care services at higher rates and at younger ages than other Australians. As a result, aged care services are planned and made available to Aboriginal people aged 50 years and above and to members of the general Australian population aged 70 years and above [7].

Largely qualitative evidence has highlighted the importance of culturally-appropriate healthcare to Aboriginal Australians [6, 8]. Aboriginal-specific services have thus become a central component of efforts to overcome these barriers and improve the health of Australian Aboriginal people [6]. There have, however, been limited attempts to demonstrate the value of these components empirically through an economics lens which poses questions about the value of such services relative to mainstream services [9–11].

This paper examines these issues through a discrete choice experiment (DCE) of participants of a culturally-specific fall prevention program. While the acceptability and effectiveness of the program has been demonstrated [12], in this study we empirically assess the preferences and willingness to pay (WTP) of participants for attending a class and the relative importance of different attributes of a class, specifically transport, cost and cultural-appropriateness to participants. An understanding of the value placed on the components of health services by the target communities and trade-offs that individuals are willing to make is vital to ensure that resources aimed at improving the health of Australia’s Aboriginal populations are used in their most effective way possible and to develop services that meet the healthcare needs of Aboriginal Australians.

### Discrete choice experiments

DCEs are based on stated-preference surveys where respondents are asked to make a series of choices between hypothetical alternatives that differ on several key attributes. DCEs are able to provide valuations of specific attributes based on individuals’ WTP, the trade-offs respondents are willing to make between attributes and overall WTP for the program in question [13]. No DCEs were found in the published health literature carried out specifically in Australian Aboriginal populations or valuing the cultural aspects of a program. Studies have attempted to value the importance of cultural factors in other sectors, for example in environmental and agricultural economics to value traditional connections to the land in Australian and Canadian Aboriginal populations [14, 15]. These studies have found differences in the preferences of Aboriginal populations to those of the non-Indigenous population, however, have emphasised the importance of extensive work in the development stages of DCE studies to ensure that the DCE is capturing the true preferences of respondents. Similarly, DCEs have been used to value components of care linked to traditional practices in other health systems around the world [13].

## Methods

A face-to-face DCE survey was administered to assess the preferences for a fall-prevention service following published guidelines for the conduct of DCEs in health [16, 17]. The study was approved by the Aboriginal Health and Medical Research Council ethics committee (1084/15).

### Participants

The DCE was administered to participants of the Ironbark program, an evidence-based, culturally-appropriate fall-prevention intervention. The intervention is a weekly group-based, balance and strength exercise class with an education component held within ‘yarning circles’ which facilitate discussion. The program targeted Aboriginal people living in the community, aged 50 years and above, however, people aged below 50 years and non-Indigenous partners or carers of participants were also able to attend in response to community demands and needs at different sites. Participants in this study were Aboriginal adults over the age of 40 years recruited through six, urban Aboriginal-specific services across New South Wales, Australia. More details on the Ironbark program have been published previously [18].

### Questionnaire

Each participant completed one questionnaire of 6 questions. Each question comprised 2 unlabelled class alternatives, A and B. For each question, respondents were asked to indicate their preference to take class A or B or no class. They were asked to assume that both classes were equally effective in preventing falls and that the classes did not differ apart from in the ways specified. The consequence of selecting no class compared with one of the classes was explained by highlighting that the participant would not receive the benefits of the class if they did not attend. A trained Aboriginal research assistant explained the questionnaire to participants, asked the questions and recorded the choices of participants.

### Attributes and levels presented

**Fig 1:**
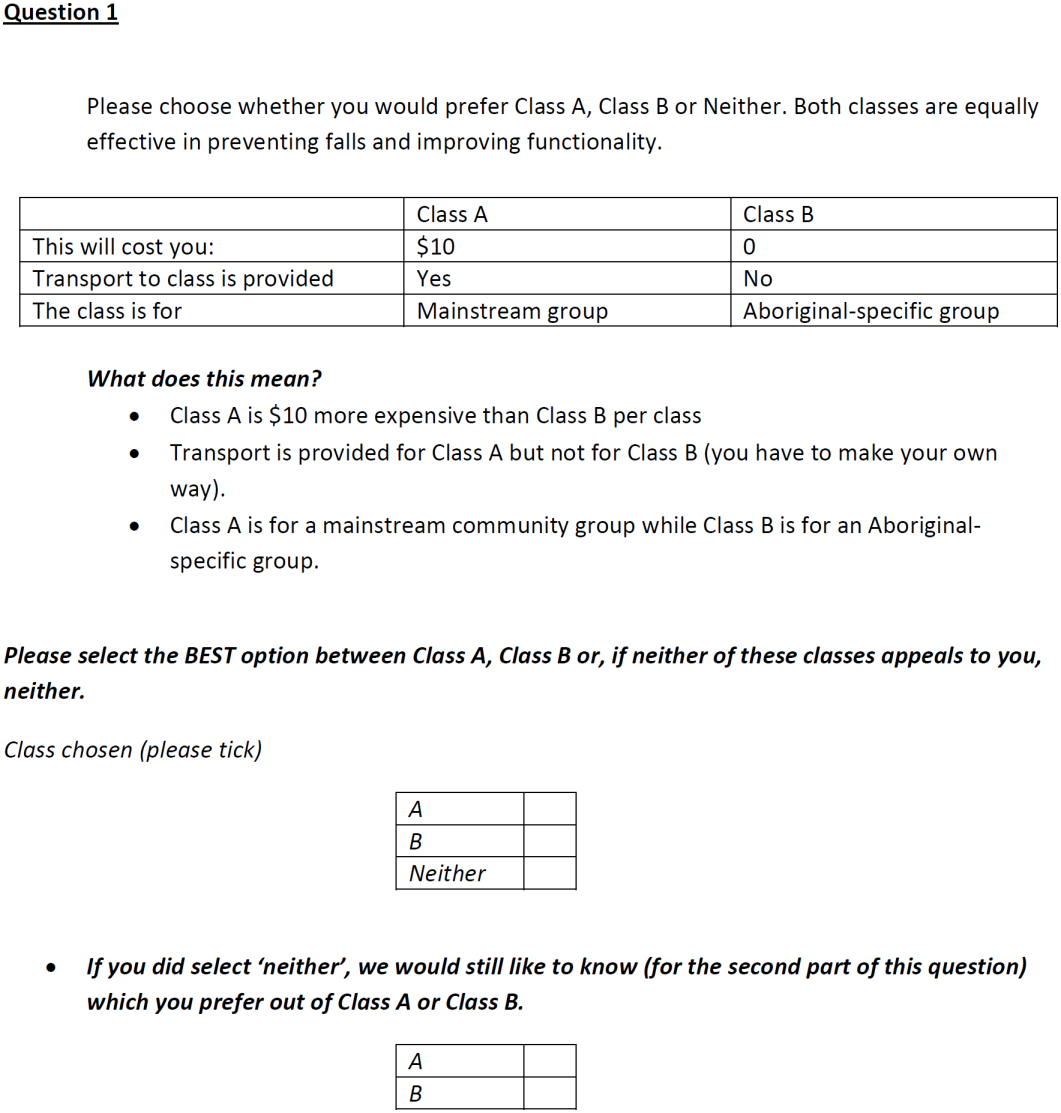
Example choice set presented to respondents.

As recommended in the DCE literature [16], attributes were developed based on qualitative work carried out in this population which identified the important factors of care to participants, literature looking at the barriers facing Aboriginal people from accessing healthcare services and discussion between the authors and the Aboriginal research assistants [19]. This process identified five factors significantly important to this population with regard to a fall-prevention class: the out-of-pocket cost of the class, whether the class was for an Aboriginal-specific population or with a mainstream population (that is a general population not specifically restricted to Aboriginal people), whether the class was delivered in a local Aboriginal community centre or other setting (such as a hospital), who delivered the class and whether transport was provided. Following this process, an initial DCE survey with attributes representing each of these components was developed with 12 questions for each participant. This DCE was piloted across three sites to a total of 39 participants to test the appropriateness of the number and levels of the attributes and assess the readability and interpretation of the questions. Feedback from participants suggested that the initial survey was too burdensome to complete in terms of the number of questions and the number of attributes presented to respondents. Through this iterative process the survey was simplified by reducing the number of attributes and levels presented to participants. In the final survey design, each class was defined by the attributes and levels outlined in Table 1. This included attributes representing the cost of the class, whether transport was provided and whether the class was for an Aboriginal-specific group. An example of the questions presented to respondents is shown in Fig 1 and the full questionnaire is provided in S1 File.

**Table 1:** Attributes and levels used in final questionnaire.

Attribute	Level
*Cost (AUD)*	$0
	$5
	$10
*Transport*	Yes
	No
*Culture*	Aboriginal-specific group
	Mainstream group

### Experimental survey design

The final survey used a d-efficient design (d-error = 0.403) based on Bayesian prior probabilities reflecting the *a priori* beliefs of the authors with regard to the expected direction of the attributes (specifically that classes that were cheaper, Aboriginal-specific and with transport provided would be preferred to other classes) using Ngene software. In line with feedback from participants and the Aboriginal research assistants during the piloting phase that the initial questionnaire was too burdensome for participants, the survey was blocked into 2 versions of 6 questions.

### Econometric and statistical analysis

DCEs are theoretically based on random utility theory where independent rational actors act to maximise their individual utility. [20] We assume that individuals choose the alternative that maximises their individual benefit or utility which depends on the attributes such that:

$$U(A\;or\;B)\;=\;\beta_{1}\;Cost\;+\;\beta_{2}\;Culture\;+\;\beta_{3}\;Transport\;+\;\varepsilon$$

U(NoClass)=0

Where:

Cost = out-of-pocket cost associated the class in Australian dollars

Culture = whether the class is Aboriginal-specific or a mainstream class

Transport = whether transport is provided

The model was estimated using panel mixed multinomial logit methods using NLOGIT 4.0. The dependent variable was the choice of the participants with a higher utility indicating a preference to attend a class compared with no class and choose one class over another. All attribute levels were effects coded with the cost variable specified as a constrained triangular distribution and the culture and transport variables specified as uniform distributions to reflect the binary nature of the attributes. A constant term was included to capture the preference of choosing a class over no class and this was specified as having a normal distribution. A panel specification was used to account for correlated choices within an individual. Limited sociodemographic information was able to be collected about participants including their age, body mass index (BMI), gender and who they lived with. The model was based on the attributes presented and interaction terms between all attributes with sociodemographic information collected. The model complexity was reduced based on the likelihood ratio test while retaining significant predictors of choice (P<0.10). Odds ratios (ORs) for choosing treatment or no treatment were derived as were participants WTP for different attributes. WTP for each attribute were estimated individually for each respondent using the *wtp* command in NLOGIT. Internal validity was tested by examining the signs and significance of parameter estimates relative to *a priori* expectations.

## Results

### General characteristics

In total 60 participants completed the final DCE questionnaire across the six sites. All participants approached to take part in this study completed the survey. The average age of participants was 64 years. The participants had an average body mass index (BMI) of 31 and 62% of participants were female. Thirty percent of the cohort lived alone, 35% lived only with a spouse and 30% lived with either their spouse and children (13%) or just their child or children (17%).

### Predictors of choice and willingness to pay

The results of the panel-mixed logit model are presented in Table 2 (the results of the multinomial logit model are presented in S1 Table). The model exhibited a good fit to the data (McFadden pseudo-R^2^ of 0.379). There was a strong underlying preference to attend a class compared to not attending as demonstrated by the significant constant term. Across the 1,080 choice observations where participants had the option to opt-out of attending a class, they chose attending a class rather than no class 1,065 (99%) of the time. Out-of-pocket costs and whether a class was Aboriginal-specific were statistically significant predictors of choice between class options with higher odds of preferring a service compared to no service with every dollar decrease in out-of-pocket cost (OR 1.19 95%CI: 1.11–1.27) and when Aboriginal-specific classes were provided (OR 1.25 95%CI: 1.00–1.55). Participants had an average willingness to pay of $1.76 (95% CI: $0.71-$2.81) for an Aboriginal-specific service relative to a mainstream population session. The provision of transport did not statistically influence the choice of service (p = 0.57). None of the interaction terms were significant predictors of the choices of participants nor improved model fit. The directions of the parameter estimates for the class characteristics regarding costs and cultural-specific care were in line with *a priori* expectations lending support to the internal validity of the model.

**Table 2:** Discrete choice experiment modeling results.

	Coefficient	Odds Ratio	p-value
Mixed Logit Model	*Pseudo-R2*	*0*.*381*	
Constant (preference for attending class as opposed to no class)	3.767		<0.001
Cost of Class	-0.172	1.188	<0.001
Aboriginal Specific Class	0.22	1.248	0.047
Transport Provided	-0.06	0.938	0.568
*Derived standard deviations of parameter distributions*			
Constant	0.172		0.041
Cost of Class	0.032		<0.001
Culture	0.107		0.047
Transport Provided	0.064		0.568

## Discussion

Since the 1970s with the introduction of the first Aboriginal Medical Service, there has been a growing recognition of the importance of culturally-specific care to the health and wellbeing of Aboriginal communities [8]. In spite of this, the value of funding a separate stream of Aboriginal-specific services when mainstream services already exist remains largely empirically untested [9, 11]. This study has empirically demonstrated the preference of this Aboriginal cohort for a culturally-specific component of a fall-prevention program. In doing so, we have demonstrated the potential of these methodologies to incorporate Aboriginal preferences and value components of services important to target populations that lie outside traditional measures of health outcomes.

Out-of-pocket costs and whether the class was Aboriginal specific were found to be significant predictors of the choices of participants. The results suggest that there was heterogeneity across the cohort preferences with significant deviations around the mean estimated coefficients. This is an important finding that needs to be further investigated and highlights the importance of community involvement and consultation in the provision of culturally-specific services to ensure that they are meeting the needs of different and potentially diverse communities. While we were not able to collect information on the income of respondents, the WTP value for a culturally-specific class of $1.76 likely reflects the constrained budgets of many in the cohort and reinforces the importance of cost as a major barrier to care facing this group. Nonetheless, given our study was examining the preference for group fall-prevention classes and the average class size in this study was ten participants, this result suggests a strong WTP for classes that are Aboriginal-specific across the group.

While we initially set out to gain information of the preferences of this cohort over a range of program characteristics, we encountered difficulties that reflected those encountered in previous work that have attempted to elicit preferences from older and otherwise marginalised populations [13, 14]. The DCE was developed utilising qualitative work, literature reviews and consultation with experts, Aboriginal researchers, community members and service providers but still required significant alteration following the piloting phase. The process highlight the need for caution in the design of DCEs to ensure that data collected reflects the true preferences of respondents. Studies in different Aboriginal populations may be able to investigate the components of a culturally-appropriate service in more detail to derive important points for service design, delivery and evaluation.

There were several further limitations to this work. The specific nature of our cohort limits the generalisability of these findings to other Aboriginal communities. Participants were recruited from urban centres and it is likely that findings and importance of the attributes, particularly the transport attribute, would be different in other populations in particular those living in more rural and remote settings. Similarly, participants were already receiving a fall-prevention intervention, it is likely that the preferences of individuals not receiving any services may be different and given the importance of engaging such populations in health services presents an important avenue for future research. We were limited by the amount of sociodemographic information that we could collect on respondents which could potentially provide greater insight into the preferences of respondents.

## Conclusion

This represents the first published DCE in the health field of an Aboriginal-only cohort. Assuming equivalent efficacy of fall-prevention programs, this study demonstrates the relative importance of overcoming two major barriers to care known to face Aboriginal Australians: out-of-pocket costs and whether the class was culturally-specific. DCEs provide a tool that can aid in valuing culturally-specific healthcare, however, care is needed in the design and use of these methods to ensure the validity of the results. Evidence demonstrating the value of such factors lying outside the traditional health-outcome framework is vital for resource-allocation decisions and to inform the design of services for Aboriginal communities.

## Supporting information

S1 Table S1 –Multinomial logistic modelling results. (DOCX)

S1 File Full questionnaire provided to respondents. (PDF)

S2 File Full dataset. (XLS)
