# Supplementary material for: Participant preferences for an Aboriginal-specific fall prevention program: Measuring the value of culturally-appropriate care
Source: PLoS One. 2018 Aug 31;13(8):e0203264. doi: 10.1371/journal.pone.0203264 (PMC6118364; doi:10.1371/journal.pone.0203264)
Supplement: S1 Table — (DOCX) [file pone.0203264.s001.docx]

| **Table S1 – Multinomial Logistic Modelling results** | **Coefficient** | **Odds Ratio** | **p-value** |
| --- | --- | --- | --- |
| Constant (preference for attending class as opposed to no class) | 3.13 |  | <0.001 |
| Cost of Class | -.163 | 1.77 | <0.001 |
| Aboriginal Specific Class | 0.14 | 1.15 | 0.0674 |
| Transport Provided | -.084 | 0.919 | 0.307 |
