# Supplementary material for: Participant preferences for an Aboriginal-specific fall prevention program: Measuring the value of culturally-appropriate care
Source: PLoS One. 2018 Aug 31;13(8):e0203264. doi: 10.1371/journal.pone.0203264 (PMC6118364; doi:10.1371/journal.pone.0203264)
Supplement: S1 File — (PDF) [file pone.0203264.s002.pdf]

PARTICIPANT RANDOMISATION NUMBER: BLOCK 1

**Thank you for agreeing to participate in this Survey Evaluation of the Ironbark Falls Prevention Program**

As part of evaluating the Ironbark project, we would like to ask you a few questions about what the most important features of the program are to you. For the next 6 questions we would like to ask you which of two hypothetical classes you would choose if you had the choice. If you would prefer to not attend either class then please select neither. If this is the case we would still like to know which class you would prefer.

Please remember that the classes are not real and your answers will not affect the Ironbark program that you are currently attending. Your responses will be used to help identify what is important about the program and how it can be made better in the future.

WHAT DO YOU NEED TO DO IN EACH QUESTION?

- Imagine that your doctor has recommended you attend an exercise class to improve your functioning and reduce your risk of falling.
- You are given a choice between Class A and Class B. Both classes are one hour long and are held once per week. Both classes are equally effective in improving your functionality and reducing your chance of having a fall.

**For each question please choose which class you would prefer: Class A, Class B or neither.**

- There are 6 questions
- Each question has a different version of Class A and Class B
- The classes are not real, you will not have to attend. We are asking you to choose the Class you would prefer if you had a choice between attending Class A, Class B or neither.

**There are no right or wrong answers!**

**PARTICIPANT RANDOMISATION NUMBER:** **BLOCK 1**

**Question 1**

Please choose whether you would prefer Class A, Class B or Neither. Both classes are equally effective in preventing falls and improving functionality.

| | Class A | Class B |
|---|---|---|
| This will cost you: | $10 | 0 |
| Transport to class is provided | Yes | No |
| The class is for | Mainstream group | Aboriginal-specific group |

***What does this mean?***

- Class A is $10 more expensive than Class B per class
- Transport is provided for Class A but not for Class B (you have to make your own way).
- Class A is for a mainstream community group while Class B is for an Aboriginal-specific group.

***Please select the BEST option between Class A, Class B or, if neither of these classes appeals to you, neither.***

*Class chosen (please tick)*

| | |
|---|---|
| *A* | |
| *B* | |
| *Neither* | |

- ***If you did select 'neither', we would still like to know (for the second part of this question) which you prefer out of Class A or Class B.***

| | |
|---|---|
| *A* | |
| *B* | |

**Question 2**

Please choose whether you would prefer Class A, Class B or Neither. Both classes are equally effective in preventing falls and improving functionality.

| | Class A | Class B |
|---|---|---|
| This will cost you: | $10 | $5 |
| Transport to class is provided | Yes | No |
| The class is for | Mainstream group | Aboriginal-specific group |

***What does this mean?***

- Class A is $5 more expensive than Class B per class
- Transport is provided for Class A but not for Class B (you have to make your own way).
- Class A is for a mainstream community group while Class B is for an Aboriginal-specific group.

***Please select the BEST option between Class A, Class B or, if neither of these classes appeals to you, neither.***

*Class chosen (please tick)*

| | |
|---|---|
| *A* | |
| *B* | |
| *Neither* | |

- ***If you did select 'neither', we would still like to know (for the second part of this question) which you prefer out of Class A or Class B.***

| | |
|---|---|
| *A* | |
| *B* | |

**Question 3**

Please choose whether you would prefer Class A, Class B or Neither. Both classes are equally effective in preventing falls and improving functionality.

| | Class A | Class B |
|---|---|---|
| This will cost you: | $10 | $5 |
| Transport to class is provided | No | Yes |
| The class is for | Aboriginal-specific group | Mainstream group |

***What does this mean?***

- Class A is $5 more expensive than Class B per class
- Transport is not provided for Class A (you have to make your own way) but is provided for Class B.
- Class A is for an Aboriginal-specific group while Class B is for a mainstream community group.

***Please select the BEST option between Class A, Class B or, if neither of these classes appeals to you, neither.***

*Class chosen (please tick)*

| | |
|---|---|
| *A* | |
| *B* | |
| *Neither* | |

- ***If you did select 'neither', we would still like to know (for the second part of this question) which you prefer out of Class A or Class B.***

| | |
|---|---|
| *A* | |
| *B* | |

**Question 4**

Please choose whether you would prefer Class A, Class B or Neither. Both classes are equally effective in preventing falls and improving functionality.

| | Class A | Class B |
|---|---|---|
| This will cost you: | 0 | $10 |
| Transport to class is provided | No | Yes |
| The class is for | Aboriginal-specific group | Mainstream group |

***What does this mean?***

- Class A is $10 cheaper than Class B per class
- Transport is not provided for Class A (you have to make your own way) but is provided for Class B.
- Class A is for an Aboriginal-specific group while Class B is for a mainstream community group.

***Please select the BEST option between Class A, Class B or, if neither of these classes appeals to you, neither.***

*Class chosen (please tick)*

| | |
|---|---|
| *A* | |
| *B* | |
| *Neither* | |

- ***If you did select 'neither', we would still like to know (for the second part of this question) which you prefer out of Class A or Class B.***

| | |
|---|---|
| *A* | |
| *B* | |

**Question 5**

Please choose whether you would prefer Class A, Class B or Neither. Both classes are equally effective in preventing falls and improving functionality.

| | Class A | Class B |
|---|---|---|
| This will cost you: | 0 | $5 |
| Transport to class is provided | No | Yes |
| The class is for | Aboriginal-specific group | Mainstream group |

***What does this mean?***

- Class A is $5 cheaper than Class B per class
- Transport is not provided for Class A (you have to make your own way) but is provided for Class B.
- Class A is for an Aboriginal-specific group while Class B is for a mainstream community group.

***Please select the BEST option between Class A, Class B or, if neither of these classes appeals to you, neither.***

*Class chosen (please tick)*

| | |
|---|---|
| *A* | |
| *B* | |
| *Neither* | |

- ***If you did select 'neither', we would still like to know (for the second part of this question) which you prefer out of Class A or Class B.***

| | |
|---|---|
| *A* | |
| *B* | |

PARTICIPANT RANDOMISATION NUMBER: BLOCK 1

**Question 6**

Please choose whether you would prefer Class A, Class B or Neither. Both classes are equally effective in preventing falls and improving functionality.

| | Class A | Class B |
|---|---|---|
| This will cost you: | 0 | $5 |
| Transport to class is provided | Yes | No |
| The class is for | Mainstream group | Aboriginal-specific group |

***What does this mean?***

- Class A is $5 cheaper than Class B per class
- Transport is provided for Class A but is not provided for Class B (you have to make your own way).
- Class A is for a mainstream community group while Class B is for an Aboriginal-specific group.

***Please select the BEST option between Class A, Class B or, if neither of these classes appeals to you, neither.***

*Class chosen (please tick)*

| | |
|---|---|
| *A* | |
| *B* | |
| *Neither* | |

- ***If you did select 'neither', we would still like to know (for the second part of this question) which you prefer out of Class A or Class B.***

| | |
|---|---|
| *A* | |
| *B* | |
